# Annual acknowledgement of reviewers

**DOI:** 10.1186/s12920-015-0081-5

**Published:** 2015-02-28

**Authors:** Tim Sands

**Affiliations:** BioMed Central, Floor 6, 236 Gray’s Inn Road, London, WC1X 8HB UK

## Abstract

The editors of BMC Medical Genomics would like to thank all our reviewers who have contributed to the journal in Volume 7 (2014).

**Djanybek Adyshev**

USA

**Ibrahim Akalin**

Turkey

**Micheala Aldred**

USA

**Tomio Arai**

Japan

**Gema Ariceta**

Spain

**Scott Auerbach**

USA

**Thomas Aune**

USA

**David Aylor**

USA

**Artem Barski**

USA

**Linnea Baudhuin**

USA

**Fatih Bayrakli**

Turkey

**Francesco Bertoni**

Switzerland

**Dwaipayan Bharadwaj**

India

**Maneesh Bhargava**

USA

**Miroslav Blumenberg**

USA

**Hoh Boonpeng**

Malaysia

**Veronica Marusa Borgonio Cuadra**

Mexico

**Pedro Borralho**

Portugal

**Jackie Boultwood**

United Kingdom

**Damien Bruno**

Australia

**Sergey Bruskin**

Russia

**Sara Busacca**

United Kingdom

**José Caldas**

Portugal

**Vincenzo Cantaluppi**

Italy

**Christopher Cardinale**

USA

**John Carlquist**

USA

**Marco Cassatella**

Italy

**Abhijit Chaudhuri**

United Kingdom

**Bor-Sen Chen**

Taiwan

**Yangchao Chen**

Hong Kong

**Si Chen**

USA

**Zheyu Chen**

China

**Wee Joo Chng**

Singapore

**Shweta Choudhry**

USA

**Yvette Conley**

USA

**Jonathan Covault**

USA

**Guoli Dai**

USA

**Ramana Davuluri**

USA

**Pasqualino De Antonellis**

Italy

**Simone De Jong**

United Kingdom

**Xavier De La Cruz**

Spain

**Eske Derks**

Netherlands

**Borislav Dimitrov**

United Kingdom

**Tamas Dolinay**

USA

**Eytan Domany**

Israel

**Paola Dongiovanni**

Italy

**Chaojun Duan**

China

**Gaurav Dugar**

Germany

**Abdou Elsharawy**

Germany

**Jonathan Lou Esguerra**

Sweden

**Francis (Biao) Feng**

Canada

**Xin Feng**

USA

**Chiara Fenoglio**

Italy

**Jia Nee Foo**

Singapore

**Michael Freeman**

USA

**Cesare Furlanello**

Italy

**Eric Gamazon**

USA

**Yuan Gao**

USA

**Joshy George**

USA

**Shanaz Ghandhi**

USA

**Sujoy Ghosh**

Singapore

**Michael Glogauer**

Canada

**Robert Greene**

USA

**Jinghua Gu**

USA

**Claudiane Guay**

Switzerland

**Michael Hackenberg**

Spain

**Dexter Hadley**

USA

**Alan H Handyside**

United Kingdom

**Steven Hart**

USA

**Yann Heuzé**

USA

**Anke Hinney**

Germany

**James Hixson**

USA

**Daniel Hyduke**

USA

**Kazushi Inoue**

USA

**Jabed Iqbal**

Singapore

**Takatsugu Ishimoto**

Japan

**Subramaniam Jayanthi**

USA

**Matlock Jeffries**

USA

**Xueqiu Jian**

USA

**Lesley Jones**

United Kingdom

**Tomasz Jurkowski**

Germany

**Susan Kadlubar**

USA

**Atsuko Kamijo-Ikemori**

Japan

**Henry Kaminski**

USA

**Do Han Kim**

South Korea

**Xiangyin Kong**

China

**Rainer Konig**

Germany

**Patrick Koty**

USA

**Thomas Laframboise**

USA

**Enzo Lalli**

France

**Rozen Le Panse**

France

**Renee Leboeuf**

USA

**Paola Lecca**

Italy

**Hane Lee**

USA

**Hyunju Lee**

South Korea

**Zhengdeng Lei**

Singapore

**Richard Lewis**

Australia

**Chunde Li**

Sweden

**Leping Li**

USA

**Chumei Li**

Canada

**Qi-Jing Li**

USA

**Can Liao**

China

**Jonathan Lippiat**

United Kingdom

**Chunyu Liu**

USA

**Li Liu**

USA

**Ying Liu**

China

**Liang Liu**

USA

**Kirk Lohmueller**

USA

**Alfons Macaya**

Spain

**Rocio Macias**

Spain

**Aaron Mackey**

USA

**Ankit Malhotra**

USA

**Adnan Mannan**

Bangladesh

**Francisco Martinez**

Spain

**Jeanne Meck**

USA

**Nan Mei**

USA

**Dan Mercola**

USA

**Seiichi Mori**

Singapore

**Wenbo Mu**

USA

**Radhakrishnan Nagarajan**

USA

**Kenneth Nephew**

USA

**Isabel Nepomuceno-Chamorro**

Spain

**Hoan Nguyen**

France

**Francisco José Ortega**

Spain

**Samir Parekh**

USA

**Hemang Parikh**

USA

**Robert Philibert**

USA

**Stefania Pizzimenti**

Italy

**Matthias Platzer**

Germany

**Igor Pogribny**

USA

**Manuel Portolés**

Spain

**Barry Powell**

Australia

**Robert Preissner**

Germany

**Todd Prickett**

USA

**Xia Pu**

USA

**Sabarinathan Radhakrishnan**

Denmark

**Bisakha Ray**

USA

**Vincent Riccardi**

USA

**Mark Robson**

USA

**Michele Rubini**

Italy

**Anguraj Sadanandam**

United Kingdom

**Noboru Sakabe**

USA

**Daniel Salomon**

USA

**George Seidel**

USA

**Abhay Sharma**

India

**Mark Shiroishi**

USA

**Michael Showe**

USA

**Antony Shrimpton**

USA

**Animesh Sinha**

USA

**Paul Skipp**

United Kingdom

**Rolf Skotheim**

Norway

**Mark Stegall**

USA

**Anat Stemmer-Rachamimov**

USA

**Alexandre Stewart**

Canada

**Petter Storm**

Sweden

**Constantine Stratakis**

USA

**Jingchun Sun**

USA

**Zhifu Sun**

USA

**Ying-Hao Sun**

China

**Xing Tang**

USA

**Cenny Taslim**

USA

**Kelsie Thu**

Canada

**Rui Tian**

USA

**Gabriele Togliatto**

Italy

**Pan Tong**

USA

**Maarten Van Den Berge**

Netherlands

**Rosa Vargas-Poussou**

France

**Patrick James Villeneuve**

Canada

**Maria Teresa Voso**

Italy

**Steve Walker**

USA

**Jing Wang**

USA

**Tzu-Hao Wang**

Taiwan

**Nanping Wang**

China

**Qianben Wang**

USA

**Kristina Warton**

Australia

**Peter White**

USA

**Rotraud Wieser**

Austria

**Matthew Wilkerson**

USA

**Ola Winqvist**

Sweden

**Kristiaan Wouters**

Netherlands

**Alexander Wyatt**

Canada

**Wenzhong Xiao**

USA

**Chengxiong Xu**

USA

**Ping Xu**

China

**Xiaojing Xu**

USA

**Yaomin Xu**

USA

**Jiekun Xuan**

USA

**Bingzhong Xue**

USA

**Junwei Yang**

China

**Joo Mi Yi**

South Korea

**Hui Yu**

USA

**Yinyin Yuan**

United Kingdom

**Ryan Yuen**

Canada

**Silvio Zaina**

Mexico

**Takeru Zama**

Japan

**Gerard Zambetti**

USA

**Zj Zang**

Singapore

**Gianluigi Zaza**

Italy

**Xianquan Zhan**

China

**Ping Zhang**

USA

**Siyuan Zheng**

USA

**Jacques Zimmer**

Luxembourg

